# Potential Roles of Mhealth for Community Health Workers: Formative Research With End Users in Uganda and Mozambique

**DOI:** 10.2196/mhealth.4208

**Published:** 2015-07-23

**Authors:** Meelan Thondoo, Daniel Ll Strachan, Maureen Nakirunda, Sozinho Ndima, Abel Muiambo, Karin Källander, Zelee Hill, The InSCALE Study Group

**Affiliations:** ^1^Institute for Global HealthUniversity College LondonLondonUnited Kingdom; ^2^Malaria ConsortiumKampalaUganda; ^3^Malaria ConsortiumMaputoMozambique; ^4^Karolinska InstitutetDeptment of Public Health SciencesStockholmSweden; ^5^Malaria ConsortiumLondonUnited Kingdom

**Keywords:** mobile phones, mHealth, community health workers, motivation, performance

## Abstract

**Background:**

Community health workers are reemerging as an essential component of health systems in low-income countries. However, there are concerns that unless they are adequately supported, their motivation and performance will be suboptimal. mHealth presents an opportunity to improve support for community health workers; however, most interventions to date have been designed through a top-down approach, rarely involve the end user, and have not focused on motivation.

**Objective:**

To use formative research to explore the views of community health workers in Uganda and Mozambique on the potential role of mHealth in their work delivering integrated community case management of children.

**Methods:**

We conducted 24 in-depth interviews and 5 focus group discussions with community health workers in Uganda and Mozambique. Data were collected on: current phone use, preferred phone and charger characteristics, and perceptions of a range of potential mHealth interventions. Interviews were conducted in the local language, were audio recorded and converted into expanded notes. Interviews were coded for key thematic areas using both deductive and inductive codes. Deductive codes included mHealth’s potential impact on motivation and performance.

**Results:**

The most salient roles of mHealth in improving performance and motivation were reducing the need for travel, improving efficiency and planning, receiving feedback and information, and improving communication with supervisors and other community health workers. This was mostly through improved voice and short message service (SMS) text communication. Specific components of mHealth interventions that participants felt could improve motivation included increasing their visibility and credibility through branding of phones; providing an SMS response to data submission; and sending SMS messages about the importance of their work and achievements, rather than just reminders or technical messages. Participants identified feasibility issues related to the language of SMS messages, network coverage, and the need for a balance between phone function and battery life. Phones with a dual SIM cards would ameliorate network problems but would reduce battery life. The provision of a solar charger was viewed as beneficial.

**Conclusions:**

Conducting formative research with end users is likely to improve mHealth interventions by: (1) identifying interventions that are likely to have the greatest impact and be the most acceptable, (2) developing salient SMS messages, and (3) identifying feasibility issues. mHealth interventions also could have an important impact on health worker motivation, which should be considered by intervention developers and in evaluations, especially as small modifications could have a significant impact. Our study suggests that using phones to improve direct communication should be considered, even when planners aim to focus on the provision of a specific application.

## Introduction

After years of relative neglect, community health workers (CHWs) are reemerging as an essential component of health systems in low-income countries [1-3]. They are central to programs such as the integrated community case management (iCCM) of childhood illness. iCCM aims to reduce child mortality by increasing the coverage of community treatment of diarrhea, pneumonia, and malaria, and the detection of malnutrition [4]. There are concerns, however, that unless CHWs are adequately supported, their retention, motivation, and performance will be suboptimal [5,6]. This could lead to a breakdown in programs, which indeed occurred in many countries in the 1980s and 1990s [7-9].

The advent of mHealth, which is the use of mobile technology to support medical and public health, and its potential to reach “new horizons for health” [10] presents an opportunity to improve support for CHWs. To date, mHealth for CHWs has primarily focused on software applications for data recording and submission, job aids to improve diagnosis and consultations, and sending and receiving short message service (SMS) text messages and reminders [11]. Stakeholders working with CHWs have highlighted the motivational potential of mobile technologies through communication with other CHWs (peer support) and with supervisors and the health system (functional support) [12], but few rigorous evaluations of mHealth have been conducted with CHWs. The limited evidence suggests that mHealth could be beneficial for performance and program efficiency [11,13]. No mHealth studies targeting CHW motivation have been published.

Previously, most CHW mHealth interventions were designed through a top-down approach, with the end user rarely involved [13]. However, research with end users is important for improving the effectiveness of intervention design, and the need to explore users’ perspectives when designing mHealth interventions is recognized [14]. In this paper we present formative research findings from Uganda and Mozambique on CHWs’ views on the potential role of mHealth in their work delivering iCCM. The data were collected as part of the formative research for the inSCALE project, which aims to test innovative approaches to improving the motivation, performance, and retention of CHWs to improve the quality and coverage of iCCM [15].

## Methods

### Data Collection

We conducted a total of 24 in-depth interviews and 5 focus group discussions with iCCM-trained CHWs on: current phone use, preferred phone and charger characteristics, and perceptions on a range of potential mHealth interventions. The potential interventions included using mobile phones to: increase contact with other CHWs, improve supervision, receive information through SMS text messages, submit data, provide treatment guidelines, and receive personalized performance-based feedback. Information on how these interventions were selected for inclusion in the interview content is available elsewhere [15].

Data were collected in January and February 2011 in the Kiboga and Hoima districts of Uganda and in March 2012 in the Massinga District and in Inhambane City in Mozambique. CHWs are called agentes polivalentes elementares (APEs) in Mozambique and village health team members (VHTs) in Uganda. In both sites, CHWs diagnose and treat childhood diarrhea, pneumonia, and malaria, but major contextual differences exist between the sites. In Mozambique, each district has 25 APEs who are each responsible for approximately 2000 people. In Uganda, VHTs are more numerous with 2-3 iCCM-trained volunteers per village, covering between 250-500 people each. In Mozambique, each supervisor oversees only 2-3 APEs. But in Uganda, 1 supervisor oversees 25-90 VHTs. In both settings, CHWs are actively selected by the communities based on their age, gender, and ability to read and write. From a financial perspective, APEs receive US $40 per month for their work, whereas VHTs are voluntary workers. The APEs are trained for 4 months while VHTs receive training for 6-11 days.

In-depth interviews and focus group discussions were conducted in the local language by trained interviewers using pretested semistructured guides. Sessions lasted between 45 minutes and 2 hours, and were audio recorded, with interviewers also taking field notes. Interviewers wrote out the findings of the in-depth interviews and focus group discussions in full in English using the expanded notes method [16]; this included recording their observations and reflections. They used the audio recordings to check completeness of their expanded notes and to add verbatim quotes. Field supervisors ensured the quality of the data through reflective meetings with interviewers and a daily review of the expanded notes.

### Recruitment

CHWs within the study districts were purposively selected from a list of iCCM trained CHWs to reflect variability in mobile network coverage and distance to supervising facility. They were approached by the interviewers and interviewed in a location of their own choosing; there were no refusals. Table 1 shows the sample size and the characteristics of participants in each setting. Sample size was determined a priori based on predictions as to when saturation in themes was likely to be reached (ie, when interviews and focus group discussions stop providing new insights into the topic). Provision was made to increase the sample size if saturation was not obtained.

**Table 1:** Sample size and characteristics of the CHWs who were selected for in-depth interviews at each site.

Characteristics		Mozambique (N=12)	Uganda (N=12)
**Age, y**			
	25-35	8	5
	36-55	4	7
**Gender**			
	Male	9	7
	Female	3	5
**Has a job in addition to being a CHW**			
	Yes	7	12
	No	5	0
**Residence**			
	Urban	0	3
	Rural	12	9
**Network availability**			
	All or most of the time	9	9
	None or only some of the time	3	3
**Distance to supervising facility**			
	<10 km	8	8
	>10 km	4	4

### Data Analysis

Data analysis started with multiple readings of the expanded notes to ensure familiarity with the data. Expanded notes were then coded for key thematic areas using both deductive and inductive methods. Inductive codes related to the potential impact of mHealth interventions on the motivation and performance of CHWs, and on the feasibility of using mobile phones. Within each inductive code, deductive coding was then conducted. Themes were then compiled into matrices to enable comparisons across participants and to identify deviant cases more easily. Data were analyzed as a team with regular reflective discussions, which included reviewing and discussing the coding frame and the emerging themes.

### Ethical Considerations

Written informed consent was obtained from all participants and the trial protocol was approved by ethical review boards at Makerere University and the Uganda National Council of Science and Technology in Uganda, the Comité Nacional de Bioética para a Saúde in Mozambique, and London School of Hygiene & Tropical Medicine Ethics Committee in the United Kingdom.

## Results

Data revealed 3 inductive themes related to the impact of mHealth interventions: performance, motivation, and the feasibility of mobile phone use.

### Performance

The main theme relating to CHW performance was the potential for mHealth interventions to ease infrastructure problems and enhance feedback. Currently, CHWs submit numerical data, such as number of children treated, using a paper-based system. This requires traveling to the supervising facility; CHWs therefore face time, cost, and transport issues. Data submission by mobile phone was perceived as having the potential to enhance performance by improving efficiency:

[I]f...we can send the statistical data via mobile phone...we would not be worried more about the transportation, travel time to go and return from the health center and the time lost in the health center in the process of data submission....

APE, 29-year-old male

CHWs also felt that a mobile phone would enable improved planning and improved referral of very sick children.

At the time of the study, CHWs received little or no feedback on their performance, which they found discouraging and felt that it hindered self-improvement: “I wonder if I’m doing well or not [in] my work...because if someone let me know that I am not doing a good work I can correct my mistakes and improve my performance” [APE, 28-year-old female]. Data submission by mobile phone was seen as a potential avenue for improved feedback, as their supervisor could use the submitted data to judge their performance and could then give feedback through a text, voice call, or face-to-face visit. CHWs stressed that feedback needs to be supportive: “Feedback should not be in form of blame but rather simple and calm advice and not given in a rude way” [VHT, 36-year-old male].

CHWs felt that receiving regular SMS text messages that provided knowledge, reminders, or advice would be beneficial for their performance. CHWs raised concerns that the content of the messages may not be relevant at the time they were received, and that message length restrictions would limit their usefulness: “The kind of information I would like to receive is anything that contributes to VHT knowledge...if they address the challenges we put across, then it would be more useful” [VHT, 52-year-old female].

CHWs felt that having treatment guidelines on their phone would benefit performance by improving their decision-making: “...when I face a difficulty in treating a patient...I can immediately check on my phone for making a better decision” [APE, 38-year-old male]. The potential availability of treatment guidelines was received less enthusiastically by CHWs than the interventions that focused on improving communication or data submission. Treatment guidelines were sometimes discussed in terms of reducing CHWs’ need to call their supervisor rather than as a new innovation: “...it would enable me to immediately check on my phone instead of calling my supervisor to ask for advice...” [APE, 28-year-old male]. There were some concerns that using guidelines would be time consuming and boring, and that unless CHWs were well trained the guidelines could be misinterpreted.

### Motivation

The main theme that emerged regarding mHealth and motivation was the ability of the phone to enhance community standing and visibility, and to improve general communication and supervision. CHWs described the potential for mobile phones to generate greater levels of community standing by increasing community trust and credibility: “A phone always has an impact on the community, it changes someone’s status and people start trusting that person” [VHT, 35-year-old male]. The degree to which a phone could increase status was linked to its perceived value:

Those modern phones are beautiful and all the people know that they are expensive. It would increase our status in the community as the people will perceive that we are recognized by the government as people who are doing a useful work....

APE, 33-year-old male

VHTs and APEs unanimously agreed that branding phones with program logos could increase visibility, status, and phone use: “Having a VHT marked phone brings you respect from the people in your community” [VHT, 36-year-old male]. Branding would also protect the phone against theft and loss.

APEs and VHTs already used their personal phones to contact each other and their supervisors, but this contact was limited by the cost of airtime. Informants felt that the provision of CHW phones and airtime would enable them to interact and support each other more frequently, and in so doing reduce stress, increase the bond felt between CHWs, allow drugs to be borrowed if a CHW ran out, and help solve problems. Many respondents felt that interactions with their peers was beneficial: “...we are able to work together as a team and solve some problems that cannot be solved by an individual...” [VHT, 35-year-old male], and expressed a collective identity and a common aim: “These interactions with other APEs are important because they avail learning from each other and makes us feel a part of 1 big family working toward the same goal...” [APE, 32-year-old female]. The potential for phones to motivate through increased contacts with other CHWs was considered greater in Mozambique, where APEs are more scattered. In Uganda, VHTs felt they could contact other VHTs in their community in person if needed.

Although most respondents wanted contact with their peers, some felt that contact with the facility was more important, especially as current contact with supervisors was often infrequent and irregular: “I like the contacts that I have with my supervisor as it allows me to improve my skills. But I need more contacts...in order to gain more experience and improve permanently my performance” [APE, 28-year-old male]. Being “on air” was perceived as allowing access to supervisors at short notice for problem-sharing, gaining information, facilitating community mobilization, and to inform supervisors about referrals of sick children: “As I live far from the health facility, being provided with a phone and to call for free would be a benefit as it would enable me to frequently be in contact with my supervisor and indeed receive support when needed” [APE, 42-year-old female]. Possible problems with voice calls or SMS included that supervisors may not be available when the CHWs called, may not call back, or may not act on reported problems, all of which were felt to be demotivating.

Other concerns were that some supervision needed to be done face-to-face, since supervisors may not get an accurate picture of what was happening over the phone and may not be able to give adequate guidance: “It is more important for the supervisor to be there physically and explain specific tasks or challenges...this cannot easily be explained on the phone” [VHT, focus group participant]. Linked to the need for face-to-face visits was a fear that phone supervision would lead to a reduction in supervisor visits, which would reduce the visibility and status of the CHWs:

I like to be visited by the health workers because it not only allows me to exchange experiences and improve my performance, but also because the community recognizes that I’m a son of the ministry of health; so I’m wondering whether the phone call from the health center could reduce such visits....

APE, 28-year-old male

Acknowledgement and feeling valued by the system was a strong motivator, and all CHWs interviewed wanted any electronic data submission to be followed by an acknowledgement: “For me, being thanked after data submission will be motivating because I’ll know that who received it recognize that I’ve performed an important task” [APE, 33-year-old male]. Participants felt that any intervention to send regular SMS text messages should not focus only on technical issues but should also stress the importance of the CHWs’ work and their achievements by, for example, telling them the number of children they have treated: “Receiving messages stressing the importance of my work in the community will mean appreciation, recognition, and indeed an encouragement to keep working” [APE, 38-year-old male].

### Feasibility of Using Mobile Phones

In both settings, CHWs felt that the impact of mHealth interventions would be undermined by patchy network coverage and system overload, but felt that this could be overcome by using phones with dual SIM cards. CHWs also expressed concern over the maintenance of the equipment and theft or damage: “What about if I lost the supplied phone or if it was stolen from me? Are you going to arrest me or ask for replacement/payment? I’m wondering because I can’t afford it...” [APE, 33-year-old male].

Despite a lack of electricity, most CHWs kept their personal phones charged: “My mobile phone is always on and with me because it belongs to me.... I know that someone can call me at any time” [APE, 28-year-old female]. Phone charging sometimes required travel and monetary costs: “In my village there are few places where one can charge the phone and it is costly.... I normally take my phone to [a] trading center..., which is about 3 km from my home” [VHT, 35-year-old female]. In response to issues of charging the phones, CHWs wanted phones that maximized battery life, even if this meant using a simple rather than a multifunctional phone. Participants felt that a solar charger would enhance their effectiveness and allow them to use more sophisticated phones with a shorter battery life: “It will save me the burden of incurring transport and charging costs” [VHT, focus group participant].

Although most CHWs had been exposed to mobile phones, they had concerns about complex phone functions or apps, but they felt they could cope if they were given training and support: “My main concern will be our ability to use these phones because it will be the first time for most/all of us to use them for this kind of work. However, with the constant training, I think we shall be able to slowly learn how to use them” [VHT, 40-year-old male]. Feasibility issues also related to language, such as sending SMS texts in the local languages, was difficult because not all CHWs spoke fluent Portuguese or English: “The challenge is...we do not understand English at the same level...” [VHT, 30-year-old female].

Themes around feasibility were also framed within the perspectives of other village and family members wanting to use the phone: “People may come asking for help to use the VHT phone and say it is a group phone and may hate you if you refuse to give it to them” [VHT, focus group participant]. There was a preference for the use of the phone to be unrestricted in terms of making calls to family and friends:

What about if there is an emergency not related to my work as APE, may I use such a phone for calling purposes? If not, what are the real advantages of having such phones...?”

APE, 46-year-old male

## Discussion

### Principal Findings

Our study found that the most salient roles of mHealth for the participants were reducing the need for travel, improving efficiency and planning, receiving feedback and information, and improving communication with supervisors and other CHWs. These reflect some of the most pressing challenges that CHWs face, and are similar to findings from a study in Senegal where phones were valued most for addressing training, stock management, reporting, and transportation challenges [17]. Despite the contextual differences between the 2 study sites, findings were surprisingly similar. For example, despite a higher supervisor-to-CHW ratio in Mozambique compared to Uganda, the desire to use mobile phones to increase and improve supervision was similar. This is likely to be related to the fact that the supervision system was not functioning optimally in either site. That despite different systems, the realities of supervision were similar.

### Limitations

A limitation of the study is that respondents were talking hypothetically, and previous studies have shown that enthusiasm for an mHealth intervention does not always correspond to uptake [14]. Participants were generally enthusiastic about the potential interventions; but this may be due to social desirability affecting reporting, which may be particularly strong when a desired commodity such as a mobile phone is being discussed. CHWs may not be in the best position to evaluate problems in their technical abilities and skills, and this study would have been strengthened by an objective assessment of CHWs’ skills.

Our findings were broadly similar in 2 very different sites. Care should be taken, however, in applying the findings to other sites as CHWs elsewhere may have different characteristics, experiences, needs, and perceptions.

### Comparison With Prior Work

Most CHW mHealth interventions to date have focused on applications for data submission, diagnosis, and SMS text message reminders [11], but our findings suggest that improving voice and text communication with other CHWs, supervisors, and health facilities could itself improve motivation, performance, and efficiency. A study in South Africa found that, although an application to improve CHW reporting of adverse events related to TB medication was not frequently used, improved text and voice communication with clients and supervisors was highly valued and enabled collaboration, reduced travel time, and made CHWs feel part of a team [14]. The potential for mobile phones to improve direct communication should be considered, even when studies or programs focus on the provision of a specific application. That said, improved communication must be 2-way, and CHWs in our study felt that if they called their supervisor and the call was not answered, or if they reported an issue and the issue was not solved, they would become demoralized. This reflects findings from Malawi on the importance of improving both communication and the outcome of the communication [18].

### Implications

By understanding CHWs’ needs, and by explicitly thinking about motivation, we were able to identify specific modifications to mHealth interventions that could improve motivation. For example, our findings suggest that intervention designers should consider how mobile phones could increase the standing, visibility, and credibility of CHWs through strategies such as clear branding of phones with the project’s logo. Other modifications that could be made to existing applications to improve motivation include providing an SMS message response to CHW data submission and sending SMS text messages about the importance of CHW work and achievements, rather than just reminders or technical messages. Data from formative research allows SMS text messages to be designed in a way that is salient to the CHWs.

Studies of the impact of mHealth on CHWs’ work have, to date, focused on measuring processes and uptake, and rigorous large-scale studies are needed with behavioral and health outcomes [11]. Our study suggests that mHealth interventions could also have an impact on motivation, and we propose that motivation should be considered during both the design and evaluation of mHealth interventions. This is supported by the “unexpected” finding from a study with HIV/AIDS community workers in Uganda where an mHealth intervention appeared to improve worker morale and job satisfaction [19]. Recently framed “pathways of research” for mHealth interventions in low-income countries do not include motivation as an outcome [20], which we feel is an important omission that should be rectified.

Our study illustrates the importance of including end-users in the design of mHealth interventions, which should become best practice for all those designing mHealth interventions. In this study, CHWs were able to identify key feasibility issues such as the language in which SMS text messages are sent and the balance between the relative importance of phone function versus battery life. We found that although poor network coverage was a reality in both settings, this could be ameliorated by providing CHWs in locations with poor coverage with a dual SIM phone. The impact of providing phones with a reduced battery life needs to be considered, and solar chargers may be a solution.

### Conclusions

There has been a call for mHealth interventions to explicitly consider theory in their design [20], using models such as the mobile phone technology acceptance model [17]. The results of the formative research presented in this paper were reviewed in light of worker performance and motivation theory, and the findings and the theory were used to design mHealth interventions in each site [15]. The use of performance and motivation theory provided a lens through which the formative research findings could be viewed and helped the team make decisions about the design of the interventions and their potential impact. We feel that the design of mHealth interventions would be strengthened by using theories that help understand performance and motivation, rather than those that focus solely on acceptance and use of the phone.

## Abbreviations

APE: agente polivalentes elementares
CHW: community health worker
iCCM: integrated community case management
SMS: short message service
VHT: village health team member

## References

[1] Bhutta Z, Lassi Z, Pariyo G, Huicho L (2010). Global experience of community health workers for delivery of health related Millennium Development Goals: A systematic review, country case studies, and recommendations for integration into national health systems.

[2] Chen L, Evans T, Anand S, Boufford JI, Brown H, Chowdhury M, Cueto M, Dare L, Dussault G, Elzinga G, Fee E, Habte D, Hanvoravongchai P, Jacobs M, Kurowski C, Michael S, Pablos-Mendez A, Sewankambo N, Solimano G, Stilwell B, de Waal A, Wibulpolprasert S (2004). Human resources for health: overcoming the crisis. Lancet.

[3] World Health Organization (2008). Task shifting: rational redistribution of tasks among health workforce teams: global recommendations and guidelines.

[4] World Health Organization, United Nations Children’s Fund (2012). WHO/UNICEF Joint Statement: Integrated Community Case Management (iCCM).

[5] Bhattacharyya K, Winch P, LeBan K, Tien M (2001). Community health worker incentives and disincentives: How they affect motivation, retention, and sustainability.

[6] Lehmann U, Sanders D (2007). Community health workers: What do we know about them?.

[7] Berman PA, Gwatkin DR, Burger SE (1987). Community-based health workers: head start or false start towards health for all?. Soc Sci Med.

[8] Gilson L, Walt G, Heggenhougen K, Owuor-Omondi L, Perera M, Ross D, Salazar L (1989). National community health worker programs: how can they be strengthened?. J Public Health Policy.

[9] Haines A, Sanders D, Lehmann U, Rowe AK, Lawn JE, Jan S, Walker DG, Bhutta Z (2007). Achieving child survival goals: potential contribution of community health workers. Lancet.

[10] World Health Organization (2011). mHealth: New horizons for health through mobile technologies.

[11] Källander K, Tibenderana JK, Akpogheneta OJ, Strachan DL, Hill Z, ten Asbroek AH, Conteh L, Kirkwood BR, Meek SR (2013). Mobile health (mHealth) approaches and lessons for increased performance and retention of community health workers in low- and middle-income countries: a review. J Med Internet Res.

[12] Strachan DL, Källander K, ten Asbroek AH, Kirkwood B, Meek SR, Benton L, Conteh L, Tibenderana J, Hill Z (2012). Interventions to improve motivation and retention of community health workers delivering integrated community case management (iCCM): stakeholder perceptions and priorities. Am J Trop Med Hyg.

[13] Braun R, Catalani C, Wimbush J, Israelski D (2013). Community health workers and mobile technology: a systematic review of the literature. PLoS One.

[14] Chaiyachati KH, Loveday M, Lorenz S, Lesh N, Larkan L, Cinti S, Friedland GH, Haberer JE (2013). A pilot study of an mHealth application for healthcare workers: poor uptake despite high reported acceptability at a rural South African community-based MDR-TB treatment program. PLoS One.

[15] Strachan DL, Källander K, Nakirunda M, Ndima S, Muiambo A, Hill Z, inSCALE study group (2015). Using theory and formative research to design interventions to improve community health worker motivation, retention and performance in Mozambique and Uganda. Hum Resour Health.

[16] Halcomb EJ, Davidson PM (2006). Is verbatim transcription of interview data always necessary?. Appl Nurs Res.

[17] Blanas DA, Ndiaye Y, MacFarlane M, Manga I, Siddiqui A, Velez O, Kanter AS, Nichols K, Hennig N (2015). Health worker perceptions of integrating mobile phones into community case management of malaria in Saraya, Senegal. Int Health.

[18] Campbell N, Schiffer E, Buxbaum A, McLean E, Perry C, Sullivan TM (2014). Taking knowledge for health the extra mile: participatory evaluation of a mobile phone intervention for community health workers in Malawi. Glob Health Sci Pract.

[19] Chang LW, Kagaayi J, Arem H, Nakigozi G, Ssempijja V, Serwadda D, Quinn TC, Gray RH, Bollinger RC, Reynolds SJ (2011). Impact of a mHealth intervention for peer health workers on AIDS care in rural Uganda: a mixed methods evaluation of a cluster-randomized trial. AIDS Behav.

[20] Chib A, van Velthoven MH, Car J (2015). mHealth adoption in low-resource environments: a review of the use of mobile healthcare in developing countries. J Health Commun.

